# *In planta* Activity of Novel Copper(II)-Based Formulations to Inhibit the Esca-Associated Fungus *Phaeoacremonium minimum* in Grapevine Propagation Material

**DOI:** 10.3389/fpls.2021.649694

**Published:** 2021-03-15

**Authors:** Enrico Battiston, Stéphane Compant, Livio Antonielli, Vincenzo Mondello, Christophe Clément, Andrea Simoni, Stefano Di Marco, Laura Mugnai, Florence Fontaine

**Affiliations:** ^1^Dipartimento di Scienze e Tecnologie Agrarie, Alimentari, Ambientali e Forestali – Sezione Patologia Vegetale ed Entomologia, Università degli Studi di Firenze, Firenze, Italy; ^2^Université de Reims Champagne-Ardenne, Unité Résistance Induite et Bioprotection des Plantes, SFR Condorcet FR CNRS 3417, Reims, France; ^3^Bioresources Unit, Center for Health and Bioresources, AIT Austrian Institute of Technology GmbH, Tulln, Austria; ^4^Dipartimento di Scienze e Tecnologie Agroalimentari, Alma Mater Studiorum – Università di Bologna, Bologna, Italy; ^5^Istituto per la Bioeconomia, Consiglio Nazionale delle Ricerche, Bologna, Italy

**Keywords:** colonization, formulation, fungicide, hydroxyapatite, nursery, plant defense

## Abstract

Grapevine trunk diseases (GTDs) are a serious and growing threat to vineyards worldwide. The need for innovative control tools persists since pesticides used against some GTDs have been banned and only methods to prevent infections or to reduce foliar symptoms have been developed so far. In this context, the application of imaging methods, already applied to study plant–microbe interactions, represents an interesting approach to understand the effect of experimental treatments applied to reduce fungal colonization, on GTD-related pathogens activity. To this aim, trials were carried out to evaluate the efficacy of copper-based treatments, formulated with hydroxyapatite (HA) as co-adjuvant with innovative delivery properties, loaded with two different copper(II) compounds (tribasic sulfate and sulfate pentahydrate), and applied to grapevine propagation material to inhibit fungal wood colonization. The treated rootstock (*Vitis berlandieri* × *Vitis riparia* cv. K5BB) and scion cuttings (*Vitis vinifera* L., cv. Chardonnay) had been inoculated with a strain of *Phaeoacremonium minimum* (*Pmi*) compared to uninoculated rootstocks. Experimental treatments were applied during the water-soaking process, comparing the copper(II) compounds pure or formulated with HA, to hydrate the cuttings. After callusing, grafted vines were grown under greenhouse conditions in a nursery and inoculated with *Pmi*::*gfp7* or with *Pmi* wild-type. Fifteen weeks post-inoculation, woody tissues close to the inoculation site were sampled to evaluate the efficiency of the treatments by studying the plant–microbe interaction by confocal laser scanning microscopy (CLSM). Copper and further elements were also quantified in the same tissues immediately after the treatments and on the CLSM samples. Finally, the grapevine defense responses were studied in the leaves of cuttings treated with the same formulations. The present investigation confirmed the relevant interaction of *Pmi* and the related transformed strain on the vascular tissues of grafted vines. Furthermore, *in vitro* assay revealed (i) the fungistatic effect of HA and the reduced effect of Cu fungicide when combined with HA. *In planta* assays showed (ii) the reduction of *Pmi* infection in propagation material treated with HA-Cu formulations, (iii) the movement of HA-Cu formulations inside the plant tissues and their persistence over time, and (iv) the plant defense reaction following the treatment with pure HA or Cu, or combined.

## Introduction

During the last decades, grapevine trunk diseases (GTDs), including Petri disease and esca complex disease (Esca), have become serious and destructive diseases in young and mature vineyards and currently represent a major issue in viticulture. The disease complexity lies notably on the plurality of the involved fungal pathogens, which are associated with specific symptomatology and epidemiology, from the nursery to the vineyard (Claverie et al., 2020) and to the relevant role of endophytic asymptomatic infections and stress factors (Hrycan et al., 2020). Since the pesticide based on sodium arsenite, which in some countries was used to reduce the damage caused by some GTDs, has been banned, new treatments have been developed (reviewed in Gramaje et al., 2018; Mondello et al., 2018b). The most promising treatments are the ones based on strains of *Trichoderma* spp. (Di Marco et al., 2004; Di Marco and Osti, 2007; Pertot et al., 2016; Berbegal et al., 2020) and *Streptomyces* spp. (Álvarez-Pérez et al., 2017; Martínez-Diz et al., 2021) and by other wound protection products (Díaz and Latorre, 2013) and foliar treatments to reduce the severity of leaf symptoms (Calzarano et al., 2014; Calzarano and Di Marco, 2018).

For all the above, the search for sustainable tools for the control of the Petri disease and Esca-related pathogens colonizing the grapevine wood, such as *Phaeoacremonium minimum* (*Pmi*) and *Phaeomoniella chlamydospora* (*Pch*), still represents an ambitious goal in established vineyards but also in grapevine propagation materials. However, the main difficulty is linked to the location of the GTD pathogens in the xylem vessels. This limits the efficiency of a control treatment as it is difficult to get it into contact with the pathogen within the vascular system. Uninjured grapevine roots and shoots can also be infected by *Pmi* (Feliciano and Gubler, 2001). This fungus colonizes the plant by occupying the intercellular spaces of the epidermis. The cortex, the pith, the rays, and xylem vessels are also colonized, but the latter more extensively (Feliciano and Gubler, 2001; Valtaud et al., 2009; Fleurat-Lessard et al., 2014).

Both *Pmi* and *Pch* pathogens are associated to structural alterations of the wood as shown by the typical Petri disease infections in young grafted vines, detectable as dark-colored phenolic compounds in xylem vessels of the rootstock, which exude out of the vessels when cut in cross sections and dark-black streaks in longitudinal cane sections (Gramaje et al., 2018). *Pmi* and *Pch* are also associated to metabolic modifications, for example, related to secretion of toxic metabolites and polypeptides, which have been detected in the xylem sap of infected vines (Bruno and Sparapano, 2006, 2007; Fleurat-Lessard et al., 2010, 2014). These results have supported, therefore, the assumption that GTD pathogens may be involved in the foliar symptom expression by transporting some signals from the colonization area, present in the trunk, to the leaves that finally express grapevine leaf stripe disease (GLSD, Abou-Mansour et al., 2004; Andolfi et al., 2011).

By defining the general basis for treatments able to control infections by the Esca-related pathogens, Roblin et al. (2019) highlighted three main key factors. First, the compounds should exhibit antimicrobial properties that inhibit fungal metabolism and, preferably, be able to kill the pathogens. Second, the compounds should be able to activate some plant defense responses as elicitors. The third major factor is related to their systemic transport throughout the plant. This model is also supported by the mechanism of action of sodium arsenite, the sole organic fungicide traditionally applied in vineyards to control Esca until it was banned in 2003 in Europe (Mazullo et al., 2000; Mondello et al., 2018b). Recent research revealed how the efficacy of sodium arsenite was associated with (i) its systemic translocation, (ii) the fungicide activity against GTD pathogens, and particularly to (iii) the effect on the vine physiology and (iv) the expression of the defense-related genes (Songy et al., 2019).

In this view, the application of a site-targeted fungicide to protect grapevine vascular tissues was investigated by several authors (Fischer et al., 2019; Wu et al., 2020). A phloem-mobile derivative from the fungicide fenpiclonil in combination with the beneficial endophyte, *Paraburkholderia phytofirmans* PsJN, to control *Neofusicoccum parvum* related to Botryosphaeria dieback was able to stimulate some plant defense responses, revealing a potential integrated control strategy against GTDs (Wu et al., 2020). Furthermore, an *in planta* targeted drug delivery showed promising results as a curative treatment against Esca. A lignin nanocarrier loaded with the fungicide pyraclostrobin injected into the grapevine trunk showed indeed an enzyme-responsive drug release after contact with Esca-associated fungi, which were then inhibited (Fischer et al., 2019). Trunk injections or endotherapy was also tested extensively in established vineyards in European countries as a possible strategy to put active substances into contact with the vascular pathogens (Mondello et al., 2018a,b). Several substances such as triazoles, fosetyl-Al, and 2-hydroxybenzoic acid were thus experimentally injected into infected grapevine trunks and tended to show interesting results by decreasing foliar symptoms expression (Calzarano et al., 2004; Darrieutort and Lecomte, 2007; Dula et al., 2007). Injection of hydrogen peroxide as well as copper nail application into infected trunks was also tested even if no real data were provided (Mondello et al., 2018a). Nevertheless, the only product currently shown to reduce the spread of Esca in vineyards is a mixture of seaweed extracts and selected minerals (Calzarano et al., 2014; Calzarano and Di Marco, 2018).

The present study arises from the need to inhibit the plant–pathogen interaction between Petri disease- and Esca-associated fungi and vascular tissues in grapevine propagation material. To this purpose and based on the interesting drug delivery properties revealed *in planta* (Battiston et al., 2018, 2019), two novel formulations based on copper(II) compounds (copper sulfate and copper tribasic sulfate) and synthetic nanostructured particles of hydroxyapatite (HA) were applied to control *Pmi*. Copper has been widely applied to control fungal infections in grapevines (La Torre et al., 2018), and previous applications for GTD management were reported by several authors (Alaniz et al., 2011; Di Marco et al., 2011; Mondello et al., 2018b). Copper oxychloride was the most effective in *in vitro*, reducing the mycelial growth and conidial germination of both *Cylindrocarpon liriodendri* and *Cylindrocarpon macrodidymum*, and *in planta*, the same compound significantly reduced the root disease severity caused by both pathogens (Alaniz et al., 2011). A further study performed by Di Marco et al. (2011) evaluated an experimental formulation based on copper oxychloride and gluconates against *Pmi* and *Pch*, revealing a significant reduction of conidial germination *in vitro* for both pathogens and the ability for such formulation to penetrate into the wood tissue *in planta*, with no significant reduction on the necrosis size caused by *Pch*. HA has been extensively studied in the medical field as a biocompatible and biomimetic material, thanks to its unique drug delivery properties, which is active on metal ions and on both organic and inorganic compounds (Suzuki et al., 1993; Kim et al., 1998; Roveri and Iafisco, 2010).

*Pmi* was reported as an interesting model pathogen in studying the plant–microbe interaction as well as for the understanding of Esca development, because *Pmi* is frequently isolated from grapevine trunks showing early Esca symptoms (Pierron et al., 2015). In trials carried out on grapevine cv. Chardonnay, *Pmi* was able to colonize the inner tissues of artificially inoculated dormant cuttings, more successfully than *Pch* does (Adalat et al., 2000). Moreover, Pierron et al. (2015) confirmed the opportunity to investigate *Pmi* in grapevine using fungal transformation with a *gfp* marked strain, detecting the pathogen colonization in the host plant using confocal laser scanning microscopy (CLSM). Previously, several marker genes have been used to study the colonization of GTD-associated fungi (Bradshaw et al., 2005; McLean et al., 2009; Mutawila et al., 2011; Landi et al., 2012). For these reasons, grafted vines of Chardonnay and *Pmi*::*gfp7* transformed strain were chosen as plant–pathogen model to perform the present study. In the same plant material, the expression of defense-related genes was analyzed in leaves to investigate the elicitor activity of the experimental cupric formulations, based on the physiological impact in grapevine of copper(II) that was extensively described by Petit et al. (2012). Additional plant defense responses occurring especially with the Esca-associated pathogen attack were carried out (Letousey et al., 2010; Lambert et al., 2013; Spagnolo et al., 2017).

Through an integrated methodology established on preliminary *in vitro* antifungal assays, imaging methods, transcriptomic analysis, and analytical techniques to detect the applied compounds, the present study aimed to verify the following objectives: (i) the *Pmi* and the related transformed strain colonization of the grafted vine and their progress along the vascular tissues during the first growing season; (ii) the fungicidal or fungistatic activity of the cupric treatments against *Pmi*, applied to the propagation material prior to grafting and *Pmi* inoculation; (iii) the activity of HA in improving the stability and persistence of the copper(II) compounds applied on grapevine woody tissues; and (iv) the activation of the GTD-related defense reactions by these treatments, applied to the leaves of the same plant material during the growing season.

## Materials and Methods

### *In vitro* Antifungal Assay

To understand the putative antifungal activity of copper(II) sulfate pentahydrate CuH_10_O_9_S (CuSPHy) and copper(II) tribasic sulfate Cu_3_H_2_O_10_S_2_ (CuTBS), pure and formulated with HA Ca_10_(PO_4_)_6_(OH)_2_, the experimental formulations (Table 1) were applied *in vitro* for the mycelial growth inhibition (GI) test according to Aqueveque et al. (2016). The strain *Pmi* CBS 100398, previously transformed with plasmid pCBCT in *Pmi*::*gfp7*, was chosen as a target pathogen associated with Petri disease and Esca, and compared to the original *Pmi* wild-type. Preliminary *in vitro* assays confirmed that they have similar behavior (data not shown). Malt Extract Agar (MEA) was autoclaved at 120°C for 15 min and fractioned under sterile conditions and amended with three different dosages of each formulation to investigate the antifungal activity of three copper(II) concentrations (0.05, 0.1, and 0.2% w/w), pure or formulated with HA (Table 1). Fifteen milliliters of such media was poured in Petri dishes (diameter 9 cm) and left to solidify. Mycelial plugs (0.7 cm diameter) of 28-day-old cultures of both *Pmi*::*gfp7* and *Pmi* wild-type were placed mycelium side down at the center of each Petri dish (three replicates per treatment). Control plates contained only MEA. Inoculated plates were incubated at 25°C in darkness. GI was calculated weekly until the 28th day post-inoculation as follows: GI = [(DC - DO)/DC] × 100, where DC is the diameter of mycelial growth in control plates and DO is the diameter of mycelial growth in treated plates.

**TABLE 1:** Experimental formulations applied **(a)** *in vitro* for the antifungal assay, as well as **(b)** *in planta* on both propagation materials as protection treatments prior to grafting and plant foliage as eliciting treatments.

**Formulation**	**HA % w/w**	**Cu(II) compound % w/w**	**Cu(II) % w/w**	**Application**	
				***In vitro (a)***	***In planta (b)***
HA:2	3	0.00	0.00	x	nd
HA	6	0.00	0.00	x	x
CuSPHy	0	21.00	5.25	x	x
CuSPHy + HA:2	3	21.00	5.25	x	nd
CuSPHy + HA	6	16.00	4.00	x	x
CuTBS	0	9.60	5.22	x	x
CuTBS + HA:2	3	9.60	5.22	x	nd
CuTBS + HA	6	7.36	4.00	x	x

*The pure copper(II) compounds and the formulations with HA were provided by the IBE Institute of the National Research Council (Bologna, Italy). The nanostructured HA (Natural Development Group Srl, Bologna, Italy) was obtained according to a patented process of synthesis (Roveri et al., 2016). Nd, not done.*

### *In planta* Plant–Pathogen Interaction Study

#### Plant Material, Pathogen Inoculation, and Experimental Treatments

Dormant rootstock (R) cuttings cv. K5BB (*Vitis berlandieri* P. × *Vitis riparia* M.) and dormant scion (S) cv. Chardonnay (*Vitis vinifera* L.) provided by the nursery Vitis Rauscedo Sca (S. Giorgio R.da, Italy) were used. The propagation material was certified according to European Directive 2005/43/EC. Furthermore, no signs of tracheomycotic infections could be detected on the base and on the top of R and S. The natural presence of *Pmi* was not previously assessed as the investigation was based on *Pmi::gfp7* transformed strain. Experimental treatments (Table 1) were applied (formulations dosage = 2% w/v) during propagation material hydration and compared to water as a control treatment, by soaking in separated batches (*n* = 6 conditions), rootstock cuttings, and scions (*n* = 15 R + 15 S × treatment and *n* = 60 R + 60 S as water treated/control material). After being grafted and callused by a nurseryman, grafted vines (*n* = 150) were planted in plastic pots and grown under greenhouse conditions (BSL-2).

Plants were inoculated when at least six leaves were fully developed, according to the method described by Pierron et al. (2015) and reviewed by Reis et al. (2019). Internodal inoculations were performed with *Pmi*::*gfp7* (*n* = 15 plants × condition) and *Pmi* wild-type (*n* = 30 control plants), by drilling (diameter 0.7 cm, depth 0.3 cm) the rootstock approximately 5 cm under the grafting point and inserting a MEA plug (diameter 0.7 cm) of 28-day-old cultures of *Pmi*::*gfp7* and *Pmi* wild-type. Negative control plants (*n* = 18 × condition) were inoculated with sterile MEA plugs.

**FIGURE 1:**
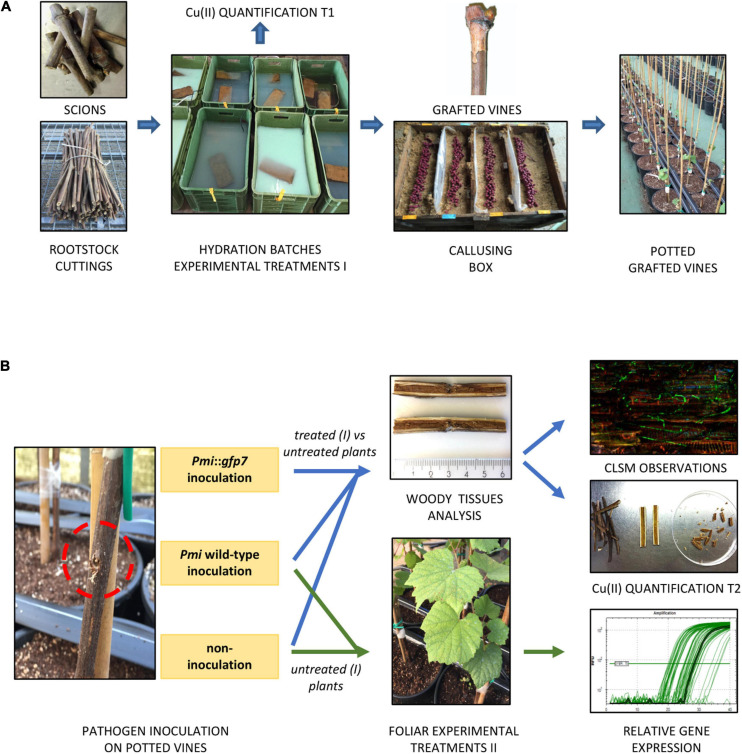
The protocol applied for the plant pathogen control study consisted in consecutive nursery operations: scion and rootstock cuttings were cut, hydrated in batches containing the experimental formulations (treatment I), grafted, callused, and potted. A first Cu(II) quantification was performed on propagation material after hydration **(A)**. Grafted potted vines were located in BSL-2 greenhouse and inoculated with (i) *Pmi::gfp7*, (ii) *Pmi* wild-type, and (iii) non-inoculated by applying a sterile MEA plug. Experimental formulations were applied to the leaves of control potted vines (treatment II), inoculated and non-inoculated, and foliar samples were finally collected for transcriptomic analysis. The rest of the potted vines were sampled at the end of the growing season to perform CLSM observations and a second Cu(II) quantification **(B)**.

The experimental treatments (Table 1) were also applied as foliar elicitors (formulations dosage = 2% w/v) by spraying the leaves of plants inoculated with *Pmi* wild-type (*n* = 3 per condition), according to the following timing: first treatment 7 weeks post-inoculation, repeated three times at 2-week intervals. Three MEA-inoculated plants were sprayed with water and used as control for transcriptomic analyses. Foliar samples were collected 8 and 24 h after the last foliar application and stored at −80°C. Plants were finally harvested 15 weeks post-inoculation and stored at 4°C until the microscopy investigation and the elementary quantification. Figure 1 describes the protocol applied to study the plant–pathogen interaction.

#### Confocal Laser Scanning Microscopy (CLSM)

The methodology used was reported by Pierron et al. (2015). Plants inoculated with *Pmi*::*gfp7* and *Pmi* wild-type were sectioned with secateurs, transversally (3 cm above and 3 cm below the inoculation site) and then longitudinally. Data result from observations (*n* = 4) of several samples (*n* = 6) for each treatment (inoculated with *Pmi*::*gfp7*) and the control plants (inoculated with *Pmi*::*gfp7* and *Pmi* wild-type), at 15 weeks post-inoculation (Figure 1B).

Images of hyphae in control or treated plants were taken using a confocal laser scanning microscope [Olympus Fluoview FV1000 with multi-line laser FV5-LAMAR-2 and HeNe(G)laser FV10-LAHEG230-2, Japan]. Observations were carried out with the 10× objective, and between 20 and 40 X, Y, Z pictures containing 20–70 scans were separately taken at 405-, 488-, and 594-nm wavelengths in blue/green/orange-red channels, respectively, with the same settings each time. Imaris software (Oxford Instruments, United Kingdom) was used by the confocal microscope to visualize 3D reconstructions. X, Y, Z pictures from different channels were then merged (RGB for red, green, and blue merging) using Image J 1.47v.

#### Inductively Coupled Plasma Optical Emission Spectrometry (ICP-OES)

Copper(II) content in the grapevine woody tissues was quantified in rootstock cuttings (*n* = 6 per condition) immediately after the treatments during hydration (T1) and on *Pmi::gfp7* inoculated plant samples (*n* = 6 per condition) harvested 15 weeks post-inoculation (T2), to evaluate the distribution and persistence of the applied copper(II). Other elements quantified were as follows: sulfur, associated with both copper(II) compounds; and calcium and phosphorus, being constituents of the applied HA. The sampled tissues at T2 correspond to the ones sampled for CLSM observations. The samples were sectioned, separating accurately the following three areas of tissues: (i) bark, (ii) vascular tissues, and (iii) parenchyma cells of pith (Figure 1B). The organic material was completely lyophilized for 48 h (Lyovapor L-300, Buchi, Swiss) and pulverized with a mixer mill (Retsch MM 400, Retsch, Germany). Afterward, 0.5 g of each sample was added to 10 ml of concentrated nitric acid and digested in a microwave oven (CEM Mars 5, Matthews, NC, United States) according to a maintenance program at 175°C × 20 min (US EPA 3050). Then, samples were brought to volume with double-distilled water and then filtered to 0.2 μm with PTFE filters and diluted 1:20 with double-distilled water. After digestion, the qualitative and quantitative determination of the elements extracted in the solution was performed using an inductively coupled plasma optical emission spectrometry (ICP-OES) instrument (Arcos-Spectro, AMETEK, Kleve, Germany).

#### Transcriptomic Analysis

This analysis was performed on leaf samples by studying a set of genes selected according to previous investigations related to GTDs and copper (Supplementary Table 1). The protocols for RNA extraction and the real-time RT-PCR analysis of gene expression were applied according to the methodology cited by Spagnolo et al. (2017). Plant RNA Purification Reagent (Thermo Fischer Scientific Inc., Waltham, MA, United States) was used to extract total RNA from 50 mg of powdered green leaf tissues treated with DNase. The quality of RNA was checked by agarose gel electrophoresis, and the quantity was determined by measuring absorbance at 260 nm. Reverse transcription was performed on 150 ng of total RNA using the Verso cDNA synthesis kit (Thermo Fischer Scientific Inc.). Real-time PCR was performed with Absolute Blue QPCR SYBR Green (Thermo Fischer Scientific Inc.) using a CFX96 thermocycler system (Bio-Rad, Hercules, CA, United States). The thermal profile was as follows: initial denaturation of 15 s at 95°C, then 40 cycles of10 s at 95°C and 45 s at 60°C (annealing/extension). Melting curve assays were performed from 65 to 95°C at 0.5°C/s. Melting peaks were visualized to check the specificity of each amplification.

### Data Analysis

Statistical analysis was performed using R Statistical Software R 4.0.2 (R Core Team, 2020). In the antifungal *in vitro assay*, measurements were repeated in three (*n* = 3) independent experiments, and linear regression analysis was applied. Analysis of variance (ANOVA) was performed on linear models to study the significance of differences (*P* ≤ 0.05) between % GI values according to HA and formulation factors. In the experiments performed *in planta*, potted grafted vines were organized as randomized complete blocks.

Assessment of *Pmi::gfp7* colonization was performed on fluorescence percentage data, considering the treatments (controls and formulations) in the absence or presence of HA, in a generalized linear model (Gamma distribution family), followed by analysis of deviance. *Post hoc* pairwise comparisons were then carried out with Estimated Marginal Means (emmeans R package).

Data of the element quantification (ICP-OES) have been logarithmically transformed in order to present all the element abundances (Ca, Cu, P, and S) regardless of the order of magnitude. Transformed data were analyzed by considering the elements together with HA, formulation, time, and tissue factors in a multivariate model. A Euclidean distance was applied on element abundances and the dissimilarity matrix analyzed by permutational multivariate ANOVA (PERMANOVA) with 9999 iterations. On the same data and using the same multivariate model, a constrained correspondence analysis (CCA) was carried out to highlight the distribution of each element in different tissues. The vectors corresponding to the abundance of each element were fit onto the CCA ordination, and the correlation of each vector to CCA axes was calculated with a permutation test (9999 iterations). A statistical test was also performed on CCA to confirm PERMANOVA results (9999 permutations). PERMANOVA, CCA ordination, and related tests were carried out with vegan R package (Oksanen et al., 2017).

Results of the relative gene expression correspond to the mean from three (*n* = 3) independent experiments. The genes analyzed were considered up- or down-regulated when changes in their expression were >2-fold or <0.5-fold, respectively. Data were also submitted to statistical analysis (ANOVA).

## Results

### *In vitro* Antifungal Assay

**FIGURE 2:**
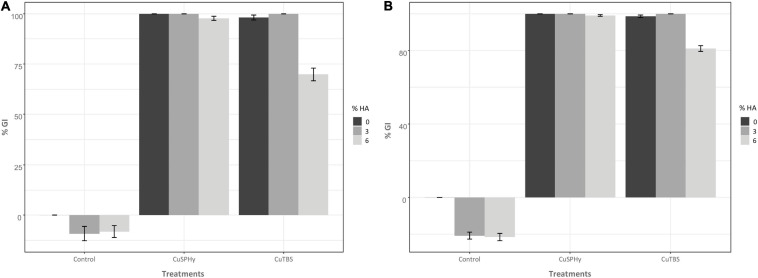
*In vitro* antifungal assay was performed comparing the mycelial growth inhibition of *Pmi*::*gfp7* **(A)** and *Pmi* wild-type strain **(B)**. In both graphics, the mycelial growth inhibition (% GI) is shown according to two different copper(II) compounds (CuSPHy and CuTBS) and to three percentages of HA (0, 3, and 6). Bar-plots show the % GI (average of three technical replicates per condition) measured four consecutive times (7, 14, 21, and 28 days post-inoculation) for each applied copper(II) concentration (0.05, 0.1, and 0.2% w/w). Bars show the standard error. A supplementary statistical analysis is reported in Supplementary Table 2.

*In vitro* mycelial growth inhibition (% GI) was measured on both strains, *Pmi*::*gfp7* and *Pmi* wild-type strains, according to the effect of the experimental formulations (Figure 2). Control plates did not reveal any inhibition of the mycelial growth of *Pmi*::*gfp7* (Figure 2A) and *Pmi* wild-type (Figure 2B), and no differences in growth between the *gfp* transformant and the wild-type were observed after fungi were grown on MEA. The lowest copper(II) concentration applied by both compounds revealed a full inhibition of the strains, already after 7 days post-inoculation (data not shown). Based on this evidence, results are presented as average of the % GI detected four consecutive times for each applied copper(II) concentration. Pure CuSPHy and CuTBS were effective in inhibiting significantly (*P* ≤ 0.05) both *Pmi* strains. Pure HA (control HA 3 and 6%) did not show any pathogen inhibition, and conversely the two HA percentages revealed a stimulant action on the mycelial growth, which was higher than the mycelial growth on both controls. The highest HA percentage significantly reduced (*P* ≤ 0.05) the antifungal activity of CuTBS on *Pmi::gfp7* and *Pmi* wild-type strains.

### *In planta* Plant–Pathogen Interaction Study

#### CLSM Images

**FIGURE 3:**
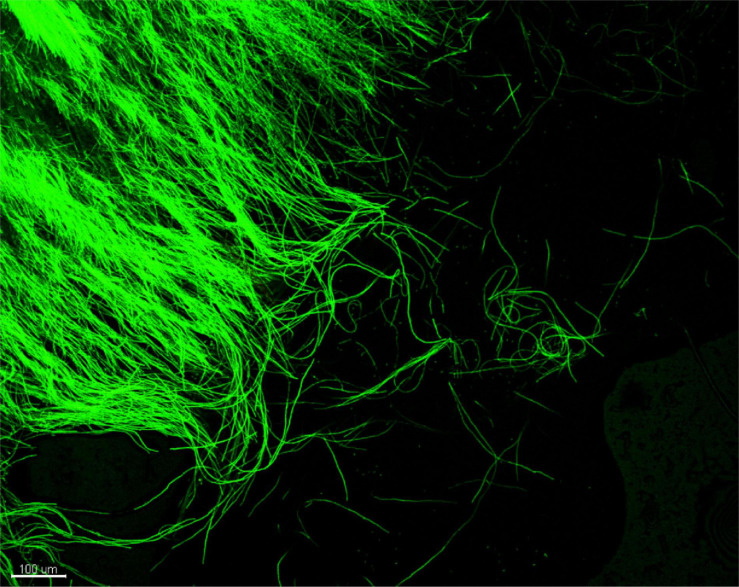
CLSM observation of *Phaeoacremonium minimum*::*gfp7* from a pure MEA culture: peripheral hyphae from plate showing strong GFP fluorescence.

Prior to inoculation, the GFP signal was verified in a pure culture of *Pmi*::*gfp7*. Figure 3 shows the intense and continuous fluorescence all over the hyphae, although punctuated distribution of the GFP signal in hyphae was occasionally observed. The *Pmi* wild-type strain did not show any green autofluorescence that could lead to a background signal (data not shown).

By processing the samples at 15 weeks post-inoculation, the inoculation site was clearly visible, as well as the bark and the vascular tissues injured by the inoculation (Figure 1B).

**FIGURE 4:**
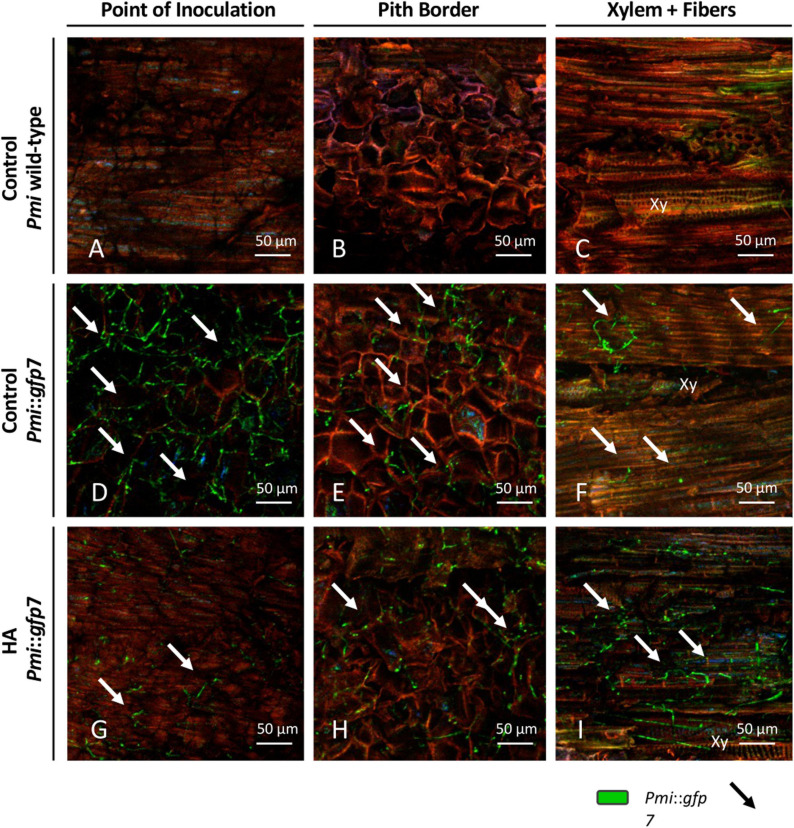
CLSM observations (four repetitions) of grafted vines samples cv. Chardonnay on Kober 5BB rootstock (six technical replicates), harvested 15 weeks post *Phaeoacremonium minimum* (*Pmi*) inoculation. Images are referred to the longitudinal section of the inoculation site (3 cm above and 3 cm below) at the rootstock level and presented according to the strain, *Pmi* wild-type **(A–C)** and *Pmi*::*gfp7* **(D–F)**, the plant tissue, point of inoculation **(A,D,G)**, pith border **(B,E,H)** and xylem with fibers **(C,F,I)**, and the HA treatment **(G,H)** *versus* the untreated controls **(A–F)**. Xy: xylem.

In Figure 4, CLSM images are presented according to (i) the *Pmi* strains, wild-type strain, and *Pmi*::*gfp7*; (ii) the plant tissue, point of inoculation, pith border, and xylem with fibers and parenchyma; (iii) and the experimental treatment, untreated control, HA, CuSPHy, CuTBS, CuSPHy + HA, and CuTBS + HA. No GFP signal was detected in the tissues of control plants inoculated with *Pmi* wild-type strain (Figures 4A–C); however, in Figure 4A, traces of the *Pmi* wild-type strain are spotted as black hyphae on the point of inoculation background. The colonization of *Pmi*::*gfp7* was revealed in control plants by the abundant fluorescence of hyphae colonizing the point of inoculation, the pith and the xylem vessels, and its surrounding material such as fibers (Figures 4D–F). There was more successful colonization of hyphae in the point of inoculation compared to other tissues (Figure 4D). Such hyphae appeared more punctuated and shorter than the ones observed from pure fungal culture (Figure 3). Hyphae of *Pmi*::*gfp7* were detected in plant material treated with HA (Figures 4G–I), especially in both xylem and the surrounding fibers and within the parenchyma, where the abundance of long hyphae confirmed the dense colonization of xylem vessel (Figure 4I). In both *Pmi*::*gfp7* control plants and HA-treated plants, the GFP signal was detected on the sample surface, suggesting a deeper colonization of the observed tissues, particularly within the pith and the fibers surrounding the xylem.

**FIGURE 5:**
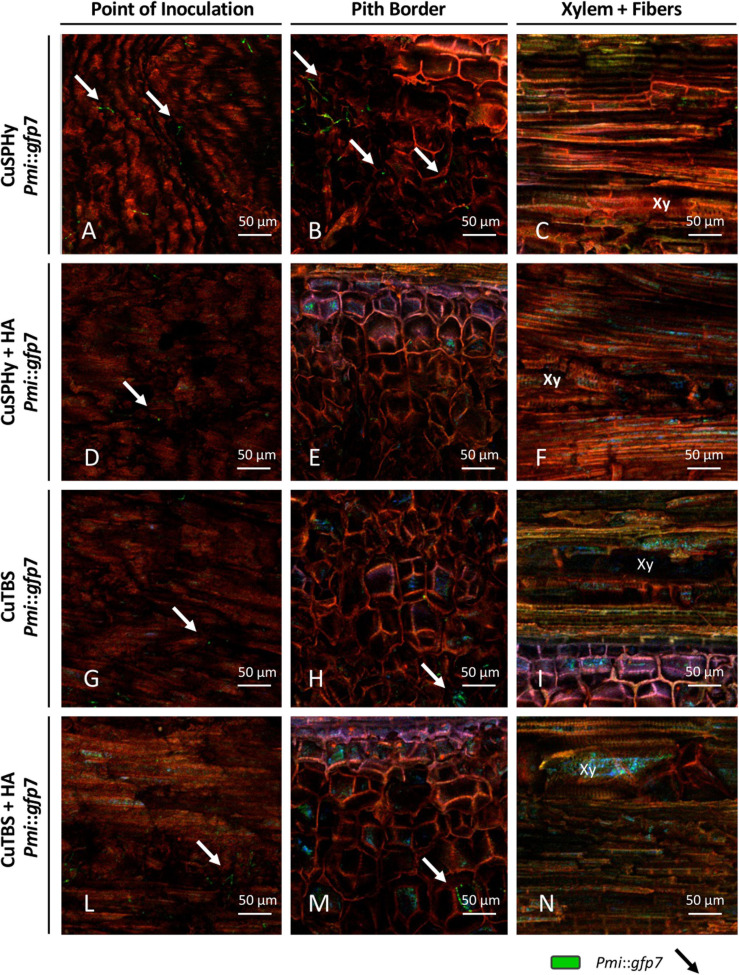
CLSM observations (four repetitions) of grafted vines samples cv. Chardonnay on Kober 5BB rootstock (six technical replicates), harvested 15 weeks post *Pmi* inoculation. Images are referred to the longitudinal section of the inoculation site (3 cm above and 3 cm below) at the rootstock level and presented according to the cupric treatments, CuSPHy **(A–C)**, CuSPHy + HA **(D–F)**, CuTBS **(G–I)**, CuTBS + HA **(L–N)**, and the plant tissue, point of inoculation **(A,D,G,L)**, pith border **(B,E,H,M)**, and xylem with fibers **(C,F,I,N)**. Xy: xylem.

GFP signal was also seen in plant material treated with the cupric formulations, revealing a different intensity and distribution of hyphae between the treatments and within the tissues surrounding the inoculation site (Figure 5). In all treatments, a very low fluorescence was detected in the inoculation point, in which punctuated hyphae emerged sparsely from the deeper tissue layers (Figures 5A,D,G,L). In the pith, only CuSPHy + HA-treated plants did not show the presence of the *Pmi*::*gfp7* (Figure 5E), while traces of the pathogen were detected in all the other treated plants (Figures 5H,M), especially in CuSPHy-treated samples (Figure 5B). In all the cupric treatments, no evidence of plant–pathogen colonization was found in the xylem vessels and the fibers surrounding the xylem (Figures 5C,F,I,N) and the very weak blue-green luminescence detected in the xylem cells was clearly associated with the background fluorescence (Figures 5C,I,N).

**FIGURE 6:**
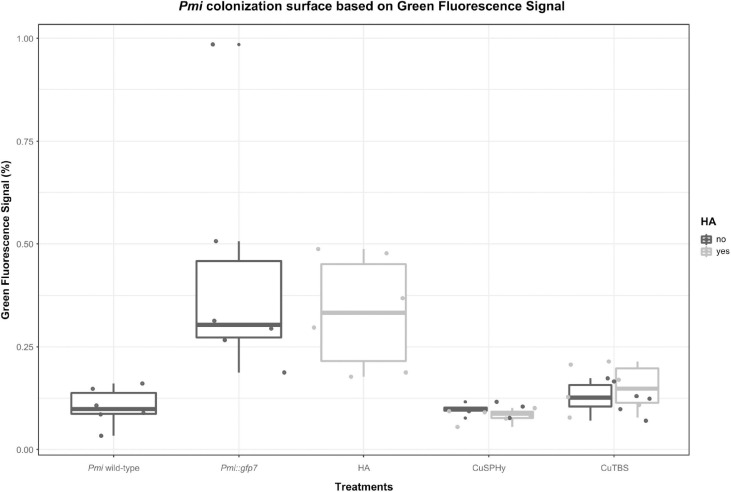
Estimation of the *Pmi*::*gfp7* colonization based on the overall fluorescent surface spotted in the CLSM images (four repetitions per six technical replicates) processed by Image J software 1.47v. Boxplots show the % of green fluorescence signal in control and treated plant samples. *Pmi* wild-type strain represents the negative control, and *Pmi*::*gfp7* represents the positive control reference. Boxplots were arranged by treatments (controls and formulations) and HA (absent, present). A supplementary statistical analysis is reported in Supplementary Table 3.

By processing the images with Image J, the area of the *Pmi*::*gfp7* colonization was estimated based on the overall fluorescent surface seen in the CLSM images. In Figure 6, such parameter is shown according to the samples related to control and treated plant material. Considering *Pmi* wild-type as negative control reference and *Pmi*::*gfp7* as positive control, the cupric treatments clearly inhibited the pathogen colonization in the tissues surrounding the inoculation site. However, HA-treated plants revealed a GFP signal percentage significantly higher, similar to the one observed in *Pmi*::*gfp7* control.

#### ICP-OES Multi-Element Determination

**FIGURE 7:**
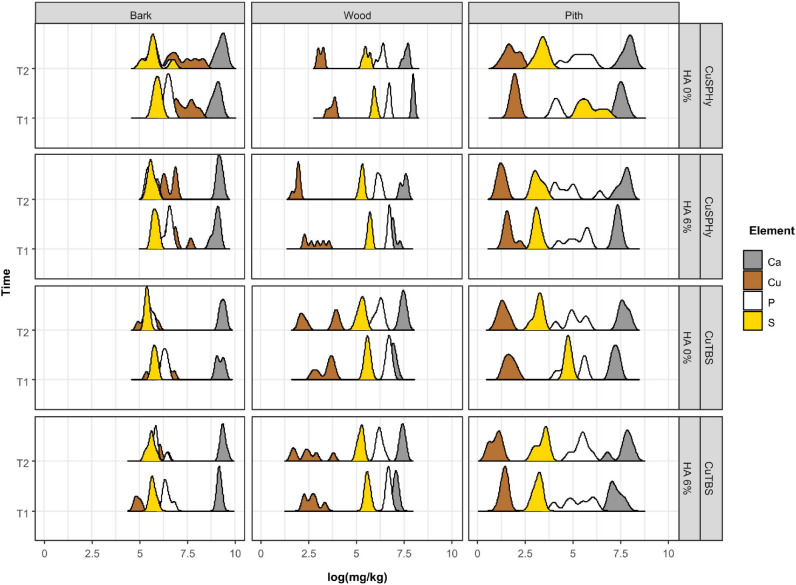
Data of the multi-elementary quantification (ICP-OES) have been logarithmically transformed to present all the elements regardless of the order of magnitude. The ridge plot shows how the abundance (mg/kg) of each element (Ca, Cu, P, and S) changes (*x*-axis) in different plant tissues (bark, wood, and pith) of six technical replicates, when different Cu formulations (CuSPHy and CuTBS) were applied alone or in combination with hydroxyapatite (HA 0%, HA 3%). Changes in element abundances were also monitored at two different time points (T1, T2), as reported in the *y*-axis. The ridge curves were plotted using ggridges and ggplot2 R packages. Differences in element abundances at each factor and interaction of factors were investigated with PERMANOVA (Supplementary Table 4).

The potential correlation between the variable plant–pathogen interaction revealed by the CLSM images and the copper(II) distribution and persistence in the same plant tissues was investigated through the ICP-OES multi-element quantification. Figure 7 shows the logarithmical transformed concentrations (mg/kg) of copper(II) and further element associated to the treatments (calcium, phosphorus, and sulfur) according to the sampled tissues (bark, wood, and pith), the detection time (post-treatment and post-harvest), and the experimental applied treatments. PERMANOVA showed that the most important factor was the tissue (pseudo-*F* = 906.21, *p* = 0.0001) explaining 84% (*R*^2^) of the overall variability, followed by time (pseudo-*F* = 44.72, *R*^2^ = 2%, *p* = 0.0001), HA (pseudo-*F* = 38.10, *R*^2^ = 1.7%, *p* = 0.0001), and formulation (pseudo-*F* = 25.70, *R*^2^ = 1.2%, *p* = 0.0001). The stability of the cupric treatments on the propagation material surface was revealed by analyzing the bark: copper(II) was particularly abundant and persistent in the CuSPHy-treated samples, followed by CuSPHy + HA, and less on both CuTBS-treated plants. In woody and pith tissues, the order of magnification changes considerably: in the vascular tissues, a relevant copper(II) persistence was found in post-harvest for all treatments, while in the pith, only a very low copper(II) accumulation was detected on CuSPHy-treated plants. Concerning the further quantified elements, calcium was found abundant in all the sampled tissues, especially in the bark, while the phosphorus and sulfur concentrations were very low in all the plant material. For such elements, no correlations with the applied treatments and the detection time emerged from the data analysis.

**FIGURE 8:**
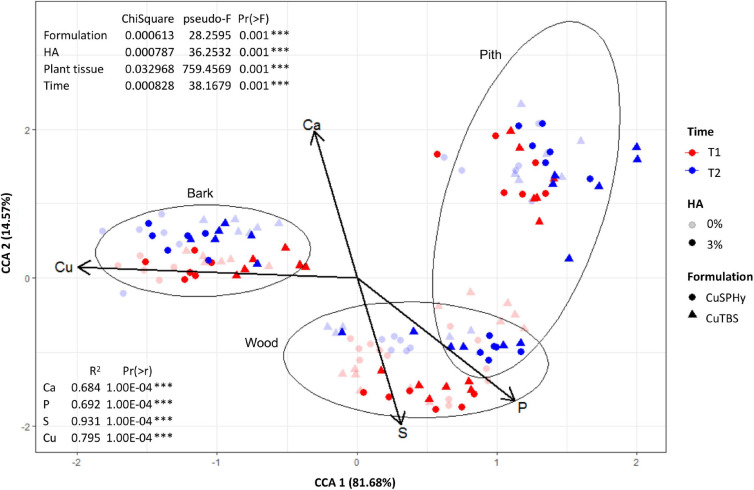
Based on the multi-elementary quantification data (ICP-OES), the constrained correspondence analysis (CCA) was performed (vegan:cca function) to highlight the distribution of each element in the sampled tissues (bark, wood, and pith), on rootstock cuttings (six technical replicates) after their treatment (T1), and on *Pmi*::*gfp7*-inoculated plant samples (six technical replicates) harvested 15 weeks post-inoculation (T2). The applied copper(II) compounds (CuSPHy and CuTBS) are spotted as different geometric shapes, with different intensities according to the HA percentage (0 and 6%) and variable color according to the sampling time (T1 and T2). Differences between tissues (bark, wood, and pith) are shown as ellipses at 95% confidence. Vectors of elements were superimposed on main CCA ordination after applying vegan:envfit function. The percentages of variation of eigenvalues are: CCA1 = 81.68% and CCA2 = 14.57%. The significance of each factor was assessed with permutation test on CCA (Supplementary Table 5).

The CCA was performed to highlight the distribution of each element in the sampled tissues at different detection times (Figure 8). By clustering the element concentrations according to the analyzed plant tissue (bark, wood, and pith), copper(II) resulted as the element mostly associated to a specific tissue, the bark, confirming the evidence shown by logarithmical transformed concentrations (Figure 7). Less specifically, phosphorus and sulfur were found to be mainly linked to the wood tissue, while calcium, being highly abundant in all the tissues, did not show any specific association. The statistical test performed on CAA (Figure 8) confirmed the PERMANOVA results (9999 permutations).

#### Relative Gene Expression

**FIGURE 9:**
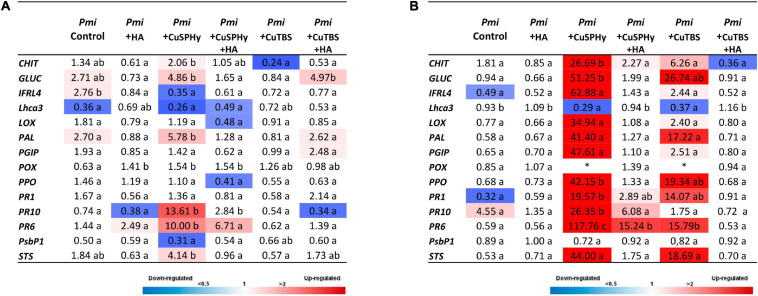
Expression levels of the selected 14 genes recorded by RT-qPCR in *Phaeoacremonium minimum* (*Pmi*) artificially inoculated grafted vines according to the applied foliar treatments (HA, CuSPHy, CuSPHy + HA, CuTBS, and CuTBS + HA) and two different detection times: 8 h **(A)** and 24 h **(B)** after the last foliar treatment. Values (the mean of three technical replicates) represent the expression levels (ΔΔC_t_) of reported conditions relatively to the control (non-inoculated and untreated plant material). Expression of a given gene was considered up- or down-regulated when the value of relative expression was > 2-fold or < 0.5-fold compared to the control, respectively. The same letter indicates no statistically significant differences for *P* ≤ 0.05 between values of different foliar treatments. (*) Gene values not available due to problems of transcription.

To study the plant response to the different experimental treatments, the expression rate of 14 defense-related genes was studied in *Pmi* artificially inoculated grafted vines at the transcriptional level by RT-qPCR. Figure 9 shows the gene expression for each condition relative to those of control (non-inoculated and water-treated). Eight hours after the last foliar treatment (Figure 9A), *Pmi* control plants revealed a weak up-regulation (>2-fold) of genes such as *PAL* (phenylpropanoides pathway), *IFRL4* (isoflavone pathway), and *GLUC* (PR-proteins family), while *Lhca3* (photosynthesis pathway) was down-regulated (<0.5-fold). A slight up-regulation of *PR6* and down-regulation of *PR10* were detected on HA-treated samples. Both copper(II) compounds revealed a variable response, especially for the formulations based on HA. CuSPHy-treated leaves showed a strong up-regulation of *GLUC, PAL*, *PR10*, *PR6*, and *STS*, while on CuSPHy + HA-treated samples, only *PR6* was up-regulated. Conversely, CuTBS + HA-treated leaves showed an up-regulation of *GLUC*, *PAL*, and *PGIP*, while the correspondent pure CuTBS treatment spotted a down-regulation of *CHIT* (defense protein). Twenty-four hours after the last foliar treatment (Figure 9B), only a high up-regulation of *PR10* was observed in *Pmi* control plants, while treatment based on HA did not show any up- or down-regulation.

On the contrary, both copper(II) compounds induced at least 10 out of the 14 targeted genes (>2-fold), with very high up-regulation values, especially in CuSPHy-treated vines (significant for *CHIT*, *GLUC*, *PPO*, *PR1*, *PR6*, and *PR10*), revealing also the high energetic cost for the plant metabolism. The correspondent formulations with HA showed the relative expression of *PR1*, *PR10*, and *PR6* for CuSPHy + HA, while the treatment based on CuTBS + HA did not reveal any gene expression but a weak repression of *CHIT* (<0.5-fold).

#### Phytotoxicity of Experimental Treatments on *V. vinifera*

*In planta* treatments allowed the visible detection of any phytotoxic effect of the tested copper-based compounds. Strong phytotoxic symptoms (typical point-like lesions) were observed on CuSPHy-treated leaves. Conversely, no phytotoxicity was observed when CuPSHy was coupled either with HA or with CuTBS-based compounds.

## Discussion

In the present study, the integrated application of an approach based on imaging–analytical–biomolecular methods was tested as a key tool to understand the effect of experimental treatments on controlling the GTD pathogen colonization and the plant responses. The results of this study revealed that (i) *Pmi* and the related transformed strain were able to colonize the grafted vine and progress along the vascular tissues during the first growing season; (ii) the applied cupric treatments showed a fungistatic effect, and for some of them, a fungicidal effect *in vitro* and *in planta* against *Pmi*; (iii) HA improved the persistence of the copper(II) compounds applied on grapevine woody tissues; and (iv) the treatments applied to the leaves during the growth season activated the GTD-related defense reactions.

### *Pmi* and the Related Transformed Strains Are Able to Colonize a Grafted Vine

Both *Pmi* and the related transformed *Pmi*::*gfp7* strains were successfully applied in *in planta* assays in the present investigation, using rootstock and scion cuttings and the resulting grafted vines, given the relevance of GTD pathogens in propagation material and young vine decline (Gramaje and Armengol, 2011; Gramaje and Di Marco, 2015; Pintos et al., 2018; Mondello et al., 2020). Thanks to CLSM investigations, the colonization of artificially inoculated *Pmi*::*gfp7* was clearly confirmed after a full vegetative season on the rootstock tissues (cv. Kober 5BB) of grafted vines (cv. Chardonnay). These observations also revealed a progressive and strong interaction with the deep layers of the cells of the pith, and along the inspected xylem vessels. The vascular tissue and pith colonization were here observed on the rootstock tissue of grafted vines. In previous research, similar results were obtained inoculating artificially GTD pathogens into the green stem of the scion (Reis et al., 2019) or inoculating artificially *Pmi* into the lignified stem of the scion, on non-grafted grapevine cuttings (Adalat et al., 2000; Pierron et al., 2015, 2016).

The method of inoculation applied in the present study was established based on the methods already described in the literature (Pierron et al., 2015, 2016; Reis et al., 2019) and the experimental context: mycelial inoculation was the effective way to inoculate *Pmi::gfp7*, verifying previously the fluorescence signal on the hyphae of the transformed strain. Inoculation with a mycelial plug was also a useful method to infect after grafting the rootstock, which are the main wood tissues of the grafted vines. However, considering the pathogenicity of the studied pathogen, a further experiment is suggested to reproduce the natural conditions under which infections occur in the nursery, namely, by inoculating the wounds with a spore suspension.

### HA Reduces the Cu Fungicide Effect When Combined in HA-Cu Formulations

The development of a novel strategy to control the grapevine–*Pmi* interaction in propagation material has been suggested by several authors, who reported that rootstock cuttings are the major source of infection by GTD pathogens in young nursery vines (e.g., Gramaje et al., 2018; Waite et al., 2018). In this context, experimental formulations based on increasing doses of copper(II) combined with two doses of HA were first tested for their activity against *Pmi*. In all the conditions established *in vitro*, all the cupric formulations provided complete inhibition of the pathogen, on both *Pmi* wild-type and *Pmi*::*gfp7* strains, thus confirming the fungicidal activity of copper(II) against Esca-associated fungi (Gramaje et al., 2009; Di Marco et al., 2011; Mondello et al., 2018b). For the HA effect when applied alone, a non-fungitoxic and stimulant activity on fungal growth was observed as previously reported in *Botrytis cinerea* (Battiston et al., 2019). Interestingly, the combination of HA-Cu showed that with the higher HA percentage, the fungicide activity of CuTBS was reduced significantly. This may be a consequence of the HA stimulant effect and of the lower copper(II) content of such formulation, which is related to the formulation stability of both CuSPHy + HA and CuTBS + HA.

On the other hand, in a previous study (Del Frari et al., 2019), copper oxychloride was associated with an increased development of the wood pathogen *P. chlamydospora* and the related brown wood streaking. A neutral effect of this copper compound on the same fungal pathogen *in planta* was reported by Di Marco and collaborators in 2011, who found a direct interaction between copper oxychloride sprayed on the bark of vines and absorbed in the xylem, and the colonization success of *P. chlamydospora*. These results, unlike the copper fungicidal effect reported in the present study, were hypothesized to be the consequence of the interaction between such copper oxychloride, the plant tissues, and the endophytes (Del Frari et al., 2019).

### HA-Cu Formulations Move Inside the Plant Tissues and Persist Over Time

According to the results obtained with *in vitro* tests, the experimental formulations with the highest HA percentage (6%) combined with the medium copper(II) concentration [0.08% of copper(II)] applied by 2% of formulation were chosen for an *in planta* assay to better understand its penetration inside the plant, the fungicide effect *in vivo*, and its effect on plant physiology. The ICP-OES multi-elementary determination thus allowed a verification of copper(II) uptake by the propagation material, prior to any natural infection during grafting, callusing, or more in general in the nursery environment, and prior to artificial inoculation with *Pmi*. As expected, immediately after the hydrating treatment in the cuttings (T1), the bark was found to be very rich in copper(II), and such concentration proved to be persistent until plant harvest (T2) for all the applied formulations, especially for CuSPHy. Despite the well-known potential phytotoxicity of copper(II) (Apel and Hirt, 2004; Yruela, 2005), such concentration did not induce any signs of phytotoxicity on the sampled tissues. The persistence of copper(II) on the cutting surface during the nursery production process might protect propagation material from the permanent risk of contamination by Esca-associated fungi colonizing natural wounds or accidentally injured tissues on such material (Waite et al., 2018). Considering the copper(II) accumulated in the inner woody tissues (both pith and vascular tissue), the higher concentration detected at T1 only results in a passive uptake that occurred during the hydration. To our knowledge, few other copper-based compounds have been tested for their ability to limit contamination by Petri disease and Esca pathogens in nurseries. One such compound is the copper bis(ethoxy−dihydroxy−diethylamino)sulfate (Cubiet^®^) tested by Gramaje and collaborators in 2009, without satisfactory results when used to eradicate or limit fungal infection during the hydration phases.

Considering the impact of copper(II) in grapevines on the resident mycobiome and the wood colonization success of some endophytes (Del Frari et al., 2019), the biological activity over time of the copper(II) applied in the present study should be investigated to understand its effect on microbial ecology and to study its efficacy in preventing conidial germination of *Pmi*, in order to avoid new infections in the wood when the copper(II) concentrations decrease.

### HA-Cu Formulations Reduce *Pmi* Infection During the Nursery Process

The CLSM images helped to verify the distribution and the interaction of *Pmi*::*gfp7* in the tissues surrounding the inoculation site, according to each applied treatment. The HA treatment had a lower pathogen presence in the point of inoculation than in the xylem, but tracking calcium and phosphorus to understand the distribution of HA, no penetration of HA inside the plant tissues was recorded. The cupric formulation treatments showed no sign of *Pmi*::*gfp7* colonization in the xylem and the surrounding fibers, confirming the significant role played by the applied copper(II) compounds as fungicide. In addition, the lower copper(II) residual presence detected at T2 is potentially responsible for the efficacy in reducing *Pmi* colonization along the vegetative season. Evaluating the combined formulations, CuSPHy + HA was the only cupric formulation that fully inhibited the *Pmi*::*gfp7* presence in the pith, while for the other copper(II)-based treatments, *Pmi*::*gfp7* was solely spotted in the pith border. However, there was no clear correlation between the *Pmi*::*gfp7* colonization and the copper(II) concentration detected at the plant harvest. As reported in a previous study on the control of *Plasmopara viticola* with similar experimental formulations (Battiston et al., 2018, 2019), CuSPHy, which is the most soluble and biologically active cupric salt, represents the most efficient compound but needs a chemical neutralization because of its high phytotoxicity. The formulation with HA, which has no fungitoxic activity, reduced the cupric fungicide effect and increased its persistence. Altogether, these results suggest that the formulation CuSPHy + HA should be further studied to better correlate the inhibition of plant–*Pmi* interaction and the persistence of copper(II) in the grapevine-treated tissues, associated to HA drug delivery properties.

### HA and Cu Alone or Combined Change Plant Responses

The experimental formulations tested in the present study showed a fungistatic effect, and for some of them, a fungicidal effect *in vitro* and *in planta*. The last step was to evaluate if such formulations could induce a response in the plant. For that, the expression of 14 targeted genes (especially related to plant defense responses) was studied. In response to *Pmi* colonization, i.e., 13 weeks after inoculation, the plant still reacts with the induction of *GLUC*, *IFRL4*, *PAL*, and *PR10*. In response to a GTD pathogen attack, a plant response was reported in most cases (Fontaine et al., 2016; Pierron et al., 2016; Pintos et al., 2018; Trotel-Aziz et al., 2019). Moreover, HA alone has no effect on plant response, and it showed no effect on *Pmi* growth, confirming its activity solely as a drug carrier (Battiston et al., 2019). The copper(II) in both formulations (CuSPHy and CuTBS) induced a few changes, an up- and down-regulation already at 8 h, while at 24 h, most of the 14 targeted genes were strongly induced. The efficiency of products based on copper(II) is recognized to be related to the effect both as fungicide and as elicitor of some plant defense responses. This copper-eliciting effect on grapevine was already observed by Aziz et al. (2006) for CuSO_4_, sprayed on leaves at concentrations > 10 μg/ml, but this was coupled to slight phytotoxicity (starting from 50 μg/ml).

More recently, other authors (Leng et al., 2015) reported, among the several effects in grapevines under Cu stress, also the induction of plant defense-related genes, such as those encoding *PR*-proteins, glucanases, and chitinases, among others. Finally, the combination HA-Cu changed the effect of Cu alone by strongly decreasing the defense induction up to no induction for CuTBS + HA formulation at 24 h, and it changed from its effect from fungicidal effect into fungistatic. The balance between the fungicidal/fungistatic effect and the plant elicitation is important for a better strategy of such formations to manage GTD pathogens in nurseries and in the future to reduce GTD expression in young and established vineyards. Moreover, these novel formulations need to take into consideration their effects on human health and in environmental protection.

## Conclusion

The novel copper(II)-based formulations, tested in this paper, propose new insights due to their lower concentration in copper(II) and their ability to move inside the plant since they were detected in different tissues of treated plants from the bark to the parenchyma cells of the pith. This characteristic will be useful to manage GTD pathogens that inhabit xylem and the surrounding tissues. In addition, some of the tested formulations may affect the growth of the pathogen and activate some plant defense responses. Novel products with all these characteristics can only be promising for a sustainable control against GTDs. However, additional work must continue to better understand the mode of action of such innovative tools and to define the best technical protocols in both nursery and vineyard.

## Data Availability Statement

The datasets presented in this study can be found in online repositories. The names of the repository/repositories and accession number(s) can be found in the article/Supplementary Material.

## Author Contributions

EB substantially conceptualized the study, designed trials, acquired data, and wrote the manuscript. SC managed the Confocal Laser Scanning Microscopy observations and the related discussion. LA performed the data analysis. VM managed the transcriptomic analysis and the related discussion. AS performed the sample preparation and the multi-element determination. CC, SD, LM, SC, and FF substantially contributed in discussing the results and revising the manuscript. All authors contributed to the article and approved the submitted version.

## Conflict of Interest

The authors declare that the research was conducted in the absence of any commercial or financial relationships that could be construed as a potential conflict of interest. The reviewer DG declared a past co-authorship with several of the authors, LA and SC, to the handling editor.

## Supplementary Material

Supplementary Table 1 Primers of genes analyzed by real-time reverse-transcription polymerase chain reaction. Thirteen genes were selected and studied according to the literature related to gene expression associated to GTDs and copper (II).

Supplementary Table 2 Statistical analysis related to the *in vitro* antifungal assay: analysis of variance (ANOVA) was performed on linear models to study the significance of differences (*P* ≤ 0.05) between % GI values according to HA and formulation factors.

Supplementary Table 3 Statistical analysis related to the estimation of the *Pmi*::*gfp7* colonizationbased on the overall fluorescent surface spotted in the CLSM images. The analysis has considered the treatments (controls, formulations) in absence or presence of HA, in a Generalized Linear Model (Gamma distribution family) followed by analysis of deviance. Post-hoc pairwise comparisons were then carried out with Estimated Marginal Means (emmeans R package).

Supplementary Table 4 Data of the element quantification (ICP-OES) have been logarithmicallytransformed in order to present all the element abundances (Ca, Cu, P e S) regardless of the order ofmagnitude. Transformed data were analysed by considering the elements together with HA, formulation,time and tissue factors in a multivariate model. A Euclidean distance was applied on element abundancesand the dissimilarity matrix analysed by permutational multivariate analysis of variances (PERMANOVA)with 9999 iterations.

Supplementary Table 5 Permutation test applied to CCA ordination of quantified element data (ICPOES). The importance of each factor (i.e. HA, Formulation, Time and Tissue) and combination thereof is reported.
