# Supplementary material for: In planta Activity of Novel Copper(II)-Based Formulations to Inhibit the Esca-Associated Fungus Phaeoacremonium minimum in Grapevine Propagation Material
Source: Front Plant Sci. 2021 Mar 15;12:649694. doi: 10.3389/fpls.2021.649694 (PMC8005723; doi:10.3389/fpls.2021.649694)
Supplement: Supplementary Table 1 — Primers of genes analyzed by real-time reverse-transcription polymerase chain reaction. Thirteen genes were selected and studied according to the literature related to gene expression associated to GTDs and copper (II). [file Table_1.pdf]

Function	Gene	Primer Sequences	GenBank accession number*
Housekeeping genes	*EF1*<br>(EF1-elongation factor)	5'-GAACTGGGTGCTTGATAGGC-3'<br>5'-AACCAAAATATCCGGAGTAAAAGA-3'	GU585871
	60SRP<br>(60S ribosomal protein L18)	5'-ATCTACCTCAAGCTCCTAGTC-3'<br>5'-CAATCTTGTCCTCCTTTCCT-3'	XM_002270599
Phenylpropanoid metabolism	*IFRL4*<br>(isoflavone reductase-like protein 4)	5'-GGATCGTGTTAATGCGGTTGA -3'<br>5'-GCCTGGCTGGACCAATGTAG -3'	BN000709
	PAL<br>(phenylalanine ammonia-lyase)	5'-TCCTCCCGGAAAACAGCTG-3'<br>5'-TCCTCCAAATGCCTCAAATCA-3'	X75967
	POX<br>(lignin-forming peroxidase)	5'-ACTGCACCAAGAAAGAGCACCAG-3'<br>5'-AGCTGTGCATGTGCCATCCCC-3'	XM_002285687.1
	STS<br>(stilbene synthase)	5'-AGGAAGCAGCATTGAAGGCTC-3'<br>5'-TGCACCAGGCATTTCTACACC-3'	FJ851185
Defense protein	*CHIT4C*<br>(chitinase class IV)	5'GGCGACGAATCCATTTATGTTA-3'<br>5'CGGAACAAGGGTTTCATAATTC-3'	AY137377
	GLUC<br>(b-1,3 glucanase)	5'-TCAATGGCTGCAATGGTGC-3'<br>5'-CGGTCGATGTTGCGAGATTTA-3'	DQ267748
	PPO<br>(polyphenol oxidase)	5'-TGGTCTTGCTGATAAGCCTAGTGA-3'<br>5'-TCCACATCCGATCGACATTG-3'	XM_002727606
	PR1<br>(pathogenesis-related protein 1)	5'-CCCAGAACTCTCCACAGGAC-3'<br>5'-GCAGCTACAGTGTCGTTCCA-3'	AJ536326
	PR6<br>(serine-protease inhibitor 6)	5'-AGGGAACAATCGTTACCCAAG-3'<br>5'-CCGATGGTAGGGACACTGAT-3'	AY156047
	PR10<br>(ribonuclease-like)	5'-GCTCAAAGTGGTGGCTTCTC-3'<br>5'- CTCTACATCGCCCTTGGTGT-3'	AJ291705
	LOX<br>(lipoxygenase)	5' -TGTTGTGTCAAGGGTCCATTC -3'<br>5'-CCCTTCTTGGCATCTCCCTTA -3'	AY159556
	PGIP<br>(polygalacturonase inhibiting prot.)	5'-CACCGGAATCTTACCACACA -3'<br>5'-GTTTGACGTCGTTGGACCTT -3'	NM_001281177.1
Photosynthesis	*Lhca3*<br>(Chlorophyll a-b binding protein)	5'- GACATACAACTACTGGGCAG-3'<br>5'-TGAGCTTCAGATCCTTGAGG-3'	AY194366
	PsbP1<br>(*oxygen-evolving enhancer PsbP subunit of photosystem I*)	5'-TGTCCCCAGCCTGTACCTTG-3'<br>5'-GCTGACGGAGATGAAGGTGG-3'	XM_002283012.4

* NCBI, National Center for Biotechnology Information.
