# Supplementary material for: In planta Activity of Novel Copper(II)-Based Formulations to Inhibit the Esca-Associated Fungus Phaeoacremonium minimum in Grapevine Propagation Material
Source: Front Plant Sci. 2021 Mar 15;12:649694. doi: 10.3389/fpls.2021.649694 (PMC8005723; doi:10.3389/fpls.2021.649694)
Supplement: Supplementary Table 2 — Statistical analysis related to the in vitro antifungal assay: analysis of variance (ANOVA) was performed on linear models to study the significance of differences (P ≤ 0.05) between % GI values according to HA and formulation factors. [file Table_2.pdf]

ANOVA Test *Pmi::gfp7*	Df	Sum Sq	Mean Sq	F value	Pr(>F)	
Formulation	1	2245.1	2245.12	900.3440	< 2.2e-16	***
HA	2	4479.3	2239.67	898.1594	< 2.2e-16	***
Concentration	2	299.3	149.63	60.0048	< 2.2e-16	***
Time	3	634.2	211.40	84.7780	< 2.2e-16	***
Formulation:HA	2	3615.7	1807.86	724.9960	< 2.2e-16	***
Formulation:Concentration	2	123.8	61.90	24.8252	5.455e-10	***
HA:Concentration	4	272.8	68.19	27.3473	< 2.2e-16	***
Formulation:Time	3	404.7	134.91	54.1027	< 2.2e-16	***
HA:Time	6	819.4	136.57	54.7663	< 2.2e-16	***
Concentration:Time	6	187.4	31.23	12.5251	2.474e-11	***
Formulation:HA:Concentration	4	113.0	28.25	11.3272	5.089e-08	***
Formulation:HA:Time	6	516.0	86.00	34.4865	< 2.2e-16	***
Formulation:Concentration:Time	6	51.9	8.66	3.4720	0.003103	**
HA:Concentration:Time	12	156.6	13.05	5.2324	2.904e-07	***
Formulation:HA:Concentration:Time	12	196.7	16.40	6.5748	2.724e-09	***
Residuals		144	359.1	2.49		

Signif. codes: 0 ‘***’ 0.001 ‘**’ 0.01 ‘*’ 0.05 ‘.’ 0.1 ‘ ’ 1

ANOVA Test *Pmi* wild-type	Df	Sum Sq	Mean Sq	F value	Pr(>F)	
Formulation	1	5268.3	5268.3	383.1764	< 2.2e-16	***
HA	2	11913.2	5956.6	433.2406	< 2.2e-16	***
Concentration	2	531.4	265.7	19.3268	3.672e-08	***
Time	4	3155.6	788.9	57.3790	< 2.2e-16	***
Formulation:HA	2	8625.0	4312.5	313.6606	< 2.2e-16	***
Formulation:Concentration	2	63.8	31.9	2.3207	0.1018695	
HA:Concentration	4	456.8	114.2	8.3062	4.674e-06	***
Formulation:Time	3	1771.3	590.4	42.9450	< 2.2e-16	***
HA:Time	6	4587.6	764.6	55.6116	< 2.2e-16	***
Concentration:Time	6	285.9	47.7	3.4663	0.0031412	**
Formulation:HA:Concentration	4	141.1	35.3	2.5662	0.0407012	*
Formulation:HA:Time	6	2475.9	412.7	30.0134	< 2.2e-16	***
Formulation:Concentration:Time	6	86.8	14.5	1.0523	0.3942225	
HA:Concentration:Time	12	219.2	18.3	1.3287	0.2083902	
Formulation:HA:Concentration:Time	12	574.0	47.8	3.4793	0.0001596	***
Residuals		144	1979.8	13.7		

Signif. codes: 0 ‘***’ 0.001 ‘**’ 0.01 ‘*’ 0.05 ‘.’ 0.1 ‘ ’ 1
