# Supplementary material for: In planta Activity of Novel Copper(II)-Based Formulations to Inhibit the Esca-Associated Fungus Phaeoacremonium minimum in Grapevine Propagation Material
Source: Front Plant Sci. 2021 Mar 15;12:649694. doi: 10.3389/fpls.2021.649694 (PMC8005723; doi:10.3389/fpls.2021.649694)
Supplement: Supplementary Table 3 — Statistical analysis related to the estimation of the Pmi::gfp7 colonizationbased on the overall fluorescent surface spotted in the CLSM images. The analysis has considered the treatments (controls, formulations) in absence or presence of HA, in a Generalized Linear Model (Gamma distribution family) followed by analysis of deviance. Post-hoc pairwise comparisons were then carried out with Estimated Marginal Means (emmeans R package). [file Table_3.pdf]

| Analysis of Deviance | Df | Deviance | Resid. Df | Resid. Dev | Pr(>Chi) | |
|---|---|---|---|---|---|---|
| NULL | | | 41 | 21.4447 | | |
| Formulation | 4 | 15.5524 | 37 | 5.8923 | < 2.2e-16 | *** |
| Formulation:HA | 2 | 0.1685 | 35 | 5.7238 | 0.5917 | |

Signif. codes: 0 ‘***’ 0.001 ‘**’ 0.01 ‘*’ 0.05 ‘.’ 0.1 ‘ ’ 1

**Post-hoc pairwise comparisons between formulations**

| | Formulation | EMM | SE | Group |
|---|---|---|---|---|
| 4 | CuSPHy | 0.09 | 0.01 | a |
| 1 | *Pmi* wild-type | 0.10 | 0.02 | ab |
| 5 | CuTBS | 0.14 | 0.02 | b |
| 3 | HA | 0.33 | 0.05 | c |
| 2 | *Pmi::gp7* | 0.43 | 0.07 | c |

Signif. codes: 0 ‘***’ 0.001 ‘**’ 0.01 ‘*’ 0.05 ‘.’ 0.1 ‘ ’ 1

**Post-hoc pairwise comparisons between formulations with no HA**

| | Formulation | EMM | SE | Group |
|---|---|---|---|---|
| 3 | CuSPHy | 0.10 | 0.02 | a |
| 1 | *Pmi* wild-type | 0.10 | 0.02 | a |
| 4 | CuTBS | 0.13 | 0.02 | a |
| 2 | *Pmi::gp7* | 0.43 | 0.08 | b |

Signif. codes: 0 ‘***’ 0.001 ‘**’ 0.01 ‘*’ 0.05 ‘.’ 0.1 ‘ ’ 1

**Post-hoc pairwise comparisons between formulations with HA**

| | Formulation | EMM | SE | Group |
|---|---|---|---|---|
| 2 | CuSPHy | 0.08 | 0.01 | a |
| 3 | CuTBS | 0.15 | 0.02 | b |
| 1 | HA | 0.33 | 0.05 | c |

Signif. codes: 0 ‘***’ 0.001 ‘**’ 0.01 ‘*’ 0.05 ‘.’ 0.1 ‘ ’ 1
