# Supplementary material for: In planta Activity of Novel Copper(II)-Based Formulations to Inhibit the Esca-Associated Fungus Phaeoacremonium minimum in Grapevine Propagation Material
Source: Front Plant Sci. 2021 Mar 15;12:649694. doi: 10.3389/fpls.2021.649694 (PMC8005723; doi:10.3389/fpls.2021.649694)
Supplement: Supplementary Table 4 — Data of the element quantification (ICP-OES) have been logarithmicallytransformed in order to present all the element abundances (Ca, Cu, P e S) regardless of the order ofmagnitude. Transformed data were analysed by considering the elements together with HA, formulation,time and tissue factors in a multivariate model. A Euclidean distance was applied on element abundancesand the dissimilarity matrix analysed by permutational multivariate analysis of variances (PERMANOVA)with 9999 iterations. [file Table_4.pdf]

PERMANOVA	Df	SumsOfSqs	MeanSqs	Pseudo-F	R^2	Pr(>F)	
Formulation	1	25.69	25.69	25.70	0.01187	0.0001	***
HA	2	38.08	38.08	38.10	0.01759	0.0001	***
Tissue	2	1811.62	905.81	906.21	0.83695	0.0001	***
Time	1	44.70	44.70	44.72	0.02065	0.0001	***
Formulation:HA	1	7.33	7.33	7.33	0.00339	0.0036	**
Formulation:Tissue	2	25.65	12.82	12.83	0.01185	0.0001	***
HA:Tissue	2	21.70	10.85	10.85	0.01002	0.0001	***
Formulation:Time	1	1.57	1.57	1.57	0.00073	0.1964	
HA:Time	1	11.54	11.54	11.54	0.00533	0.0006	***
Tissue:Time	2	19.14	9.57	9.57	0.00884	0.0001	***
Formulation:HA:Tissue	2	3.43	1.72	1.72	0.00158	0.1530	
Formulation:HA:Time	1	4.21	4.21	4.21	0.00195	0.0294	*
Formulation:Tissue:Time	2	3.29	1.64	1.64	0.00152	0.1803	
HA:Tissue:Time	2	22.23	11.12	11.12	0.01027	0.0001	***
Formulation:HA:Tissue:Time	2	4.43	2.22	2.22	0.00205	0.0906	.
Residuals	120	119.95	1.00		0.05541		
Total	143	2164.56			1.00000		

Signif. codes: 0 '***' 0.001 '**' 0.01 '*' 0.05 '.' 0.1 ' ' 1
