# Supplementary material for: In planta Activity of Novel Copper(II)-Based Formulations to Inhibit the Esca-Associated Fungus Phaeoacremonium minimum in Grapevine Propagation Material
Source: Front Plant Sci. 2021 Mar 15;12:649694. doi: 10.3389/fpls.2021.649694 (PMC8005723; doi:10.3389/fpls.2021.649694)
Supplement: Supplementary Table 5 — Permutation test applied to CCA ordination of quantified element data (ICPOES). The importance of each factor (i.e. HA, Formulation, Time and Tissue) and combination thereof is reported. [file Table_5.pdf]

Permutation test on CCA	Df	ChiSquare	Pseudo-F	Pr(>F)	
Formulation	1	0.000613	28.2595	0.001	***
HA	1	0.000787	36.2532	0.001	***
Tissue	2	0.032968	759.4569	0.001	***
Time	1	0.000828	38.1679	0.001	***
Formulation:HA	1	0.000025	1.1615	0.272	
Formulation:Tissue	2	0.000553	12.7502	0.001	***
HA:Tissue	2	0.000765	17.6173	0.001	***
Formulation:Time	1	0.000008	0.3701	0.657	
HA:Time	1	0.000230	10.5988	0.002	**
Tissue:Time	2	0.000717	16.5220	0.001	***
Formulation:HA:Tissue	2	0.000089	2.0450	0.106	
Formulation:HA:Time	1	0.000114	5.2526	0.010	**
Formulation:Tissue:Time	2	0.000094	2.1625	0.093	.
HA:Tissue:Time	2	0.000720	16.5762	0.001	***
Formulation:HA:Tissue:Time	2	0.000057	1.3062	0.281	.
Residual	120	0.002605			

Signif. codes: 0 ‘***’ 0.001 ‘**’ 0.01 ‘*’ 0.05 ‘.’ 0.1 ‘ ’ 1
