# Supplementary material for: In planta Activity of Novel Copper(II)-Based Formulations to Inhibit the Esca-Associated Fungus Phaeoacremonium minimum in Grapevine Propagation Material
Source: Front Plant Sci. 2021 Mar 15;12:649694. doi: 10.3389/fpls.2021.649694 (PMC8005723; doi:10.3389/fpls.2021.649694)
Supplement: Supplementary file 6 [file Presentation_1.pdf]

SUPPLEMENTAL TABLE 1S | Primers of genes analyzed by real-time reverse-transcription polymerase chain reaction. Thirteen genes were selected and studied according to the literature related to gene expression associated to GTDs and copper(II).

SUPPLEMENTAL TABLE 2S | Statistical analysis related to the *in vitro* antifungal assay: analysis of variance (ANOVA) was performed on linear models to study the significance of differences ($P \leq 0.05$) between GI% values according to HA and formulation factors.

SUPPLEMENTAL TABLE 3S | Statistical analysis related to the estimation of the *Pmi::gfp7* colonization based on the overall fluorescent surface spotted in the CLSM images. The analysis has considered the treatments (controls, formulations) in absence or presence of HA, in a Generalized Linear Model (Gamma distribution family) followed by analysis of deviance. Post-hoc pairwise comparisons were then carried out with Estimated Marginal Means (emmeans R package).

SUPPLEMENTAL TABLE 4S | Data of the element quantification (ICP-OES) have been logarithmically transformed in order to present all the element abundances (Ca, Cu, P e S) regardless of the order of magnitude. Transformed data were analysed by considering the elements together with HA, formulation, time and tissue factors in a multivariate model. A Euclidean distance was applied on element abundances and the dissimilarity matrix analysed by permutational multivariate analysis of variances (PERMANOVA) with 9999 iterations.

SUPPLEMENTAL TABLE 5S | Permutation test applied to CCA ordination of quantified element data (ICP-OES). The importance of each factor (*i.e*. HA, Formulation, Time and Tissue) and combination thereof is reported.
